# Comprehensive evaluation of the underground space resources in Xianyang city

**DOI:** 10.1038/s41598-023-44657-8

**Published:** 2023-10-13

**Authors:** Shifeng Li, Zenglin Hong, Xuping Xue, Xiaofeng Liu, Wei Shi

**Affiliations:** 1 School of Land Engineering, Chang’an University, Xi’an, 710054 China; 2 Shaanxi Institute of Geological Survey, Xi’an, 710054 China; 3 Shaanxi Hydrogeology Engineering Geology and Environment Geology Survey Center, Xi’an, 710054 China

**Keywords:** Sustainability, Environmental social sciences

## Abstract

With the rapid development of urbanization and the sharp increase in population, urban land is becoming increasingly scarce. The efficient and reasonable development of the underground space is a crucial way to solve the problem of urban diseases, and comprehensive evaluation of urban underground space resources is an important basic task to achieve reasonable planning of the underground space. Adopting Xianyang city as an example, in this paper, we comprehensively evaluated the underground space resources in the main urban area and established evaluation models for the amount of resources available for development, development difficulty, potential value, and comprehensive quality of the underground space. Evaluation indicators, including urban environmental constraints, geological conditions, socioeconomic conditions and many other factors, were determined. With the use of the method of item-by-item elimination of restrictive elements and the analytic hierarchy process for determining the weight of each evaluation index, GIS technology was used to calculate and evaluate the underground space resources (0–30 m) in the main urban area of Xianyang city that could be reasonably developed, as well as the corresponding development difficulty and potential value, and we obtained the underground space that could be reasonably developed under different types of land use in the main urban area of Xianyang city on the basis of the resource quantity and comprehensive quality evaluation results. The results showed that in terms of quantity, the amount of underground space available for development in the main urban area of Xianyang city accounts for approximately 25.11% of the total development amount, and the underground space that could be developed and utilized is approximately 82.3 km^2^. The underground space resources that could be developed within a 30 m depth interval in the main urban area reached 2.465 billion m^3^, accounting for approximately 79.5% of the total shallow underground space resources, and the potential for development and utilization is enormous. In terms of the comprehensive quality, the highest comprehensive quality level of shallow underground resources is located in the core areas along Renmin Road, Weiyang Road, and Century Avenue, with an area of 21.52 km^2^, and the highest comprehensive quality level of subshallow underground resources is located along Renmin Road and Weiyang Road, with an area of 4.37 km^2^. The evaluation results could provide high reference value for urban development planning and underground space development and utilization in Xianyang.

## Introduction

In recent years, with the rapid development of extensive cities in China, urbanization has provided people the convenience and efficiency of modern life, while problems such as a large population coupled with a small land area, a shortage of resources, traffic congestion, urban waterlogging, and environmental pollution have become increasingly prominent, seriously affecting the quality of life of people and the sustainable development of cities. The efficient and rational development and utilization of the underground space could resolve these urban diseases. Scientific assessment of the amount of available resources, development difficulty, potential value and comprehensive quality of the urban underground space is an important basic task to realize rational urban underground space planning^1,2^.

At present, many scholars have evaluated urban underground space resources from different perspectives. To evaluate the amount of resources available for the development and utilization of the underground space in Tianjin, Wang Yongli^3^ used a GIS-fuzzy comprehensive evaluation model. Through evaluation, a database of underground space resources was established, and a distribution map of underground space resources was generated. The resources available for the development and utilization of the underground space were characterized and measured. To evaluate the difficulty of underground space development, Li Pengyue et al.^4^ established a geological suitability evaluation model for the development and utilization of urban underground space resources based on their collaborative development and utilization and the comprehensive response to geological environmental problems. Analytical methods and fuzzy comprehensive evaluation methods were used to evaluate the difficulty of developing underground space resources in Chengdu. To evaluate the development potential of the urban underground space, Liu Jianan et al.^5^ adopted Wuhan city as an example, comprehensively considered three factors, namely, geological environment suitability, existing construction restrictions, and social and economic benefits, to construct an evaluation index system, and used the analytic hierarchy process for calculating a comprehensive underground space index of the development potential and conducting potential zoning by systematically considering ecological protection and cultural relic protection restrictions in Wuhan city. This index system and potential zones provided a scientific reference for the subsequent development and utilization of the urban underground space. Wang Xia et al.^6^ used the multilevel grey evaluation method to construct an evaluation index system for the development potential of urban underground space resources and evaluated the development potential of the underground space in Nanjing. This evaluation method required few samples and could be used to address the information inconsistency in the evaluation process. The problem of completeness and uncertainty provides new ideas for the evaluation of the development potential of urban underground space resources. To explore comprehensive quality assessment of underground space resources, Wu Xinzhen^7^ used GIS technology and a fuzzy comprehensive evaluation method to assess the suitability and potential development value of underground space resources in Wuhu city, conducted a comprehensive quality assessment of underground space resources, and delineated underground space zoning management and control strategies. The evaluation method and results guided the subsequent execution of urban underground space comprehensive utilization planning.

There are many ways to evaluate urban underground space resources. The more mature methods include the analytic hierarchy process^8–12^, fuzzy comprehensive evaluation method^13–15^, extension evaluation method^16,17^, negative list method^18^, variable fuzzy set theory^19^, multilevel grey evaluation method^6^, fuzzy c-means clustering algorithm^20^, random forest algorithm^21^, or a combination or improvement of the above methods^4,7,22–30^. The above methods have been widely employed to evaluate the amount of resources available for development, development difficulty, potential value and quality of underground space resources. However, there are certain limitations when used for the comprehensive evaluation of underground space resources from multiple perspectives, such as insufficient selection of indicator factors and weak hierarchy. Therefore, in this paper, we used multiple methods and combined models to comprehensively evaluate urban underground space resources. Since there are many factors that affect the development of underground space resources and each evaluation factor exhibits different attributes, indicators, and qualitative and quantitative standards, this task is complex and multilayered. Therefore, it is impractical to use a deterministic model to calculate the weights of evaluation factors.

Based on the above analysis, in this paper, we constructed a comprehensive evaluation index system for urban underground space resources, evaluated the underground space development resource quantity, development difficulty, and potential value and established comprehensive quality assessment models. Evaluation indicators, including urban environmental constraints, geological conditions, socioeconomic conditions and many other factors, were determined. The method of item-by-item elimination of restrictive factors was used to evaluate the amount of underground space resources available for development, and the analytic hierarchy process was used to determine the weight of each evaluation index. The evaluation results were visualized through GIS technology to comprehensively evaluate underground space resources in the study area.

With the rapid economic development and population growth in the main urban area of Xianyang city, which is an important development city in Shaanxi Province, urban land resources are in short supply, and the contradiction between humans and land is prominent. The large-scale development and utilization of the urban underground space are inevitable during sustainable urban development. Therefore, it is of practical significance to comprehensively evaluate the underground space resources in Xianyang city.

## Description of the study area

**Figure 1:**
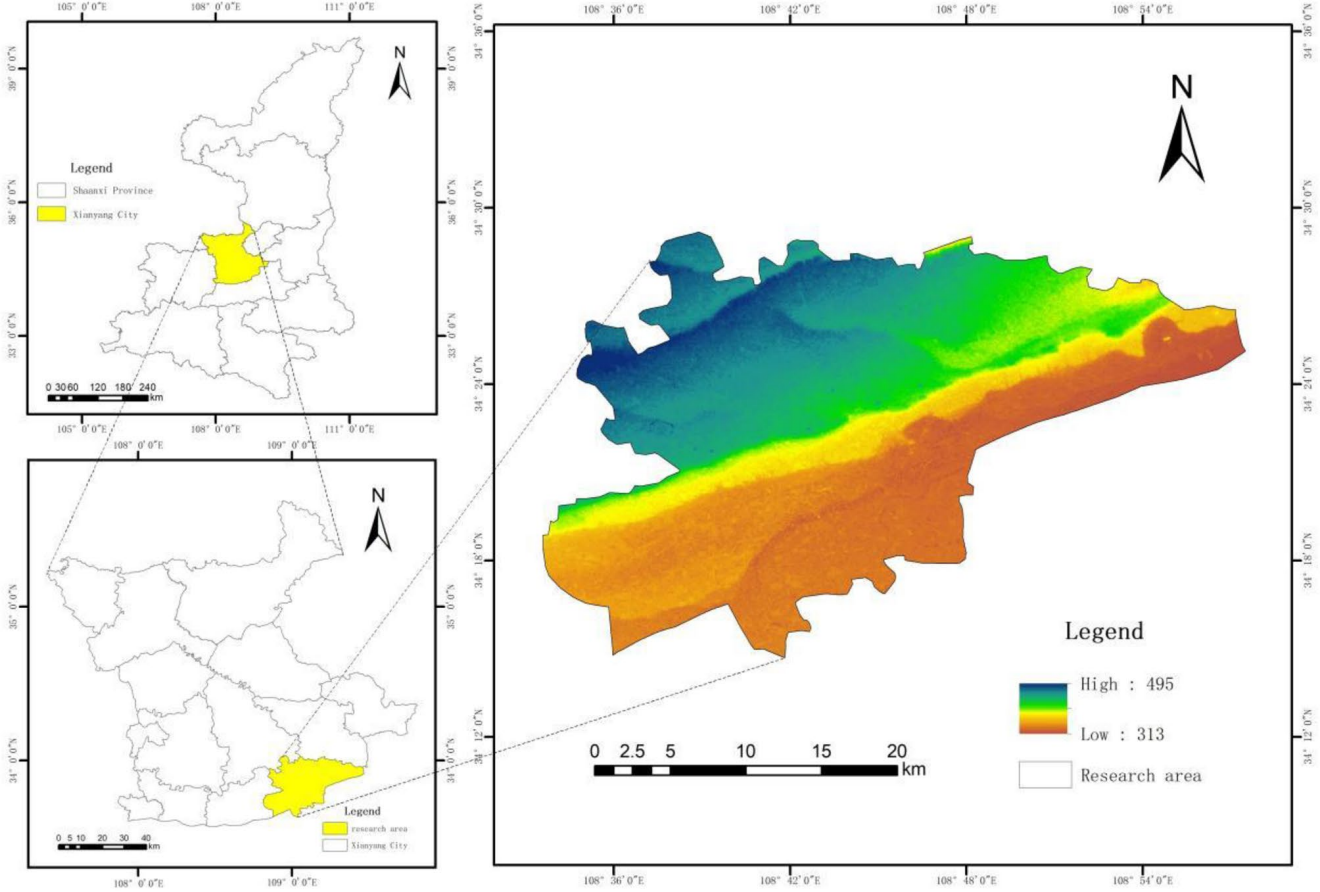
Overview of the location of the study area.

The scope of the study area is the main urban area of Xianyang city. Xianyang city is located in the hinterland of the Guanzhong Plain in Shaanxi Province, bordering Xi'an to the east, Chang'an and Hu County to the south, Yangling, Fufeng, and Linyou to the west, and the Tongchuan and Qingyang and Pingliang areas in Gansu Province to the north. Moreover, the terrain is high in the north and low in the south, with low mountains in the north and plains in the south. From north to south, the landforms include medium and low mountains, loess hills, loess plateaus and Weihe River terraces, as shown in Fig. 1.

The scope of the study area is the main urban area of Xianyang city. Xianyang city is located in the hinterland of the Guanzhong Plain in Shaanxi Province, bordering Xi’an in the east, Chang’an Hu County in the south, Yangling, Fufeng and Linyou in the west, and the Tongchuan and Qingyang and Pingliang areas in Gansu in the north. The terrain is high in the north and low in the south, with low mountains in the north and plains in the south. From north to south, the landforms include medium and low mountains, loess hills, loess plateaus and Weihe terraces, as shown in Fig. 1. The strata are Quaternary, Palaeogene and Neogene strata, and the lithology within 0–30 m mainly includes clayey soil, sand, gravel soil, loess, and gravel. The groundwater type in the area is karst groundwater. As of 2019, there were 245 underground spaces in the main urban area of Xianyang city, with a total development area of 1.549 million m^2^, and the per capita underground space area was approximately 2.2 m^2^. The type of underground space utilization mainly entailed underground garages, accounting for 82% of the underground space development ratio in Xianyang city. Moreover, underground space development was limited to shallow depths, with a scattered distribution, thus lacking horizontal connectivity, while a network could not be formed.

## Study methods and evaluation process

### Evaluation method

**Figure 2:**
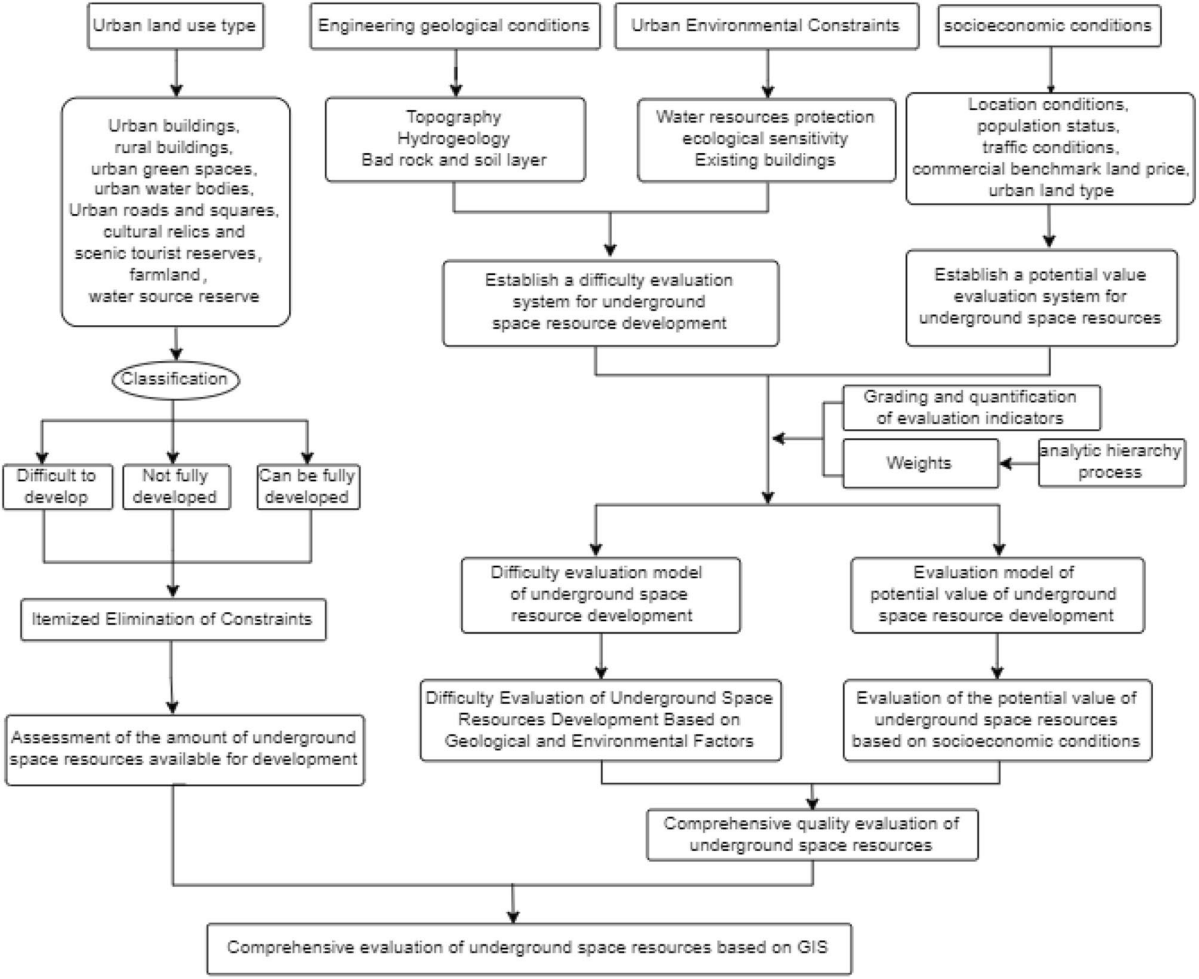
Evaluation method structure diagram.

To comprehensively evaluate underground space resources, the quantity, quality, and favourable and restrictive factors of resource development of underground space resources should be assessed within a certain scope. In this paper, the underground space resources available for development in the study area were evaluated by the method of eliminating influencing factors one by one. Because there are many factors affecting the comprehensive quality of underground space resources and these factors are mutually restricted, interrelated, multilevel and complex, the analytic hierarchy process was used for evaluation, and the ArcGIS spatial analysis function was employed to visualize the evaluation results. A flow chart of the evaluation process is shown in Fig. 2.

### Evaluation model

#### Estimation model for the underground space resources available for development

The degree of exploitability of underground space resources is affected by many factors. The most effective method for estimating the amount of underground space resources potentially available for development is item-by-item elimination of restrictive factors. First, we must determine the types of urban land use and apply remote sensing methods to obtain land use type data such as ground buildings, water bodies, green spaces, and farmland^31,32^. Then, we must divide the study area into difficult-to-develop areas, insufficient-to-develop areas, and fully developable areas according to the development and utilization of resources (Table 1). Unexploitable areas can be obtained by superimposing the underground space range under each constraint factor, and the amount of unexploitable resources can be deducted from the total amount of underground space resources to obtain the amount of resources available for development^33^. The calculation model of urban underground space resources under each land type is summarized in Table 2. The model can be expressed as:

1 $$ {\varvec{V}} = {\varvec{V}}_{{\varvec{n}}} - \bigcup\limits_{{{\varvec{i}} = 1}}^{k} {{\varvec{V}}_{{\varvec{i}}} } $$

where V_n_ denotes the natural underground space resources in the planning area, V denotes the potential exploitable space resources, and V_i_ (i = 1, 2… k) is the amount of space resources under restrictive factors such as geological hazards, water bodies and developed underground spaces.

**Table 1:** Factors influencing the exploitable degree of underground space resources.

Degree of development	Factor
Difficult to develop	Water resources and spring vein reserves, active faults, ground fissures (extremely prone areas and highly prone areas), cultural relic reserves, and ecological reserves
Not fully exploited	Green spaces, water bodies, mountains, ground buildings, roads and interchanges, and developed underground spaces
Can be fully exploited	Farmland and new planned construction areas

**Table 2:** Amount of urban underground space resources under the various land use types.

Land use type	Capacity
Urban buildings	According to the type of building, 6 m, 15 m and 30 m are deducted for low-rise, mid-rise and high-rise buildings, respectively
Rural buildings	Mostly low-rise buildings, deducting a depth of 6 m
Urban green spaces	3 m after deducting the vegetation protective layer
Urban water bodies	Deduct 10 m
City roads and squares	Deduct 5 m of the overlay
Farmland	Deduct 3 m of the overlay
Cultural relics, scenic tourism reserves	Deduct all depths within 30 m
Areas prone to geological hazards	After deducting the full depth of 30 m in the area prone to geological disasters
Water source reserves	Deduct the full depth of the protected area of first-class water sources
Developed underground spaces	Deduct the depth of influence of 15 m

#### Evaluation model for the difficulty of underground space resource development

Engineering geological conditions and urban environmental constraints are the main factors affecting the difficulty of underground space resource development, and the development difficulty can be evaluated according to shallow (0 ~ 15 m) and subshallow (15 ~ 30 m) areas on the basis of various factors affecting different spatial domains. Regarding the main urban area of Xianyang city, the influencing factors of the difficulty of underground space resource development are shown in Tables 3 and 4, and the evaluation models can be expressed as follows:

2 $$ S_{d} = \alpha \sum\limits_{j = 1}^{m} {w_{j} } \sum\limits_{k = 1}^{n} {w_{jk} u_{jk} } $$

where u_jk_ is the value of the kth indicator in the jth indicator layer; w_jk_ is the weight of the kth indicator in the jth indicator layer; w_j_ is the weight of the jth topic layer; S_d_ is the development difficulty evaluation result; and α is the difficulty reduction factor. Usually, the deeper the excavation is, the more difficult the construction. According to past experience, shallow and subshallow layers are considered, and the reduction coefficients are 1 and 0.9, respectively.

**Table 3:** Weight of the resource development difficulty index of the shallow (0 ~ 15 m) underground space.

Theme layer	Weight	Indicator layer	Weight	Weighted general order
Topography	0.108	Ground elevation	0.5	0.054
		Terrain slope	0.5	0.054
Bedrock and soil layer	0.234	Stratigraphic lithology	0.5	0.117
		Sand liquefaction	0.5	0.117
Hydrogeology	0.283	Water content	0.6	0.170
		Dive depth	0.4	0.113
Water resource protection	0.202	Secondary water source	1	0.202
Ecological sensitivity	0.173	Ecological spatial sensitivity	1	0.173

**Table 4:** Weight of the resource development difficulty index of the subshallow (15 ~ 30 m) underground space.

Theme layer	Weight	Indicator layer	Weight	Weighted general order
Bedrock and soil layer	0.159	Stratigraphic lithology	1	0.159
Hydrogeology	0.382	Water content	0.6	0.229
		Dive depth	0.4	0.153
Water resource protection	0.316	Secondary water source	1	0.316
Existing buildings	0.143	Depth of influence of existing buildings	1	0.143

#### Evaluation model for the potential value of underground space resource development

The potential value of underground space resources can be determined via the comprehensive assessment of the probability of obtaining the expected levels of social, economic and environmental benefits at the urban location of underground space resources. According to the evaluation of the Xianyang area, through a large number of investigations, the potential value evaluation index of underground space resource development was finally determined (Table 5). With the use of the multifactor weighted average method, a potential value assessment model for underground space resources based on socioeconomic conditions was established as follows:

3 $$ S_{e} = \alpha \sum\limits_{j = 1}^{m} {w_{j} u_{j} } $$

where u_j_ is the value of the jth indicator, w_j_ is the weight of the jth index, S_e_ is the potential value, and α is the value reduction coefficient. The deeper the excavation is, the lower the potential value of development.

**Table 5:** Potential value index weight of underground space resources.

Theme layer	Weight	Indicator layer	Weight
Socioeconomic conditions	1	Location conditions	0.233
		Demographic situation	0.192
		Traffic conditions	0.230
		Commercial benchmark land price	0.190
		Urban land use type	0.155

#### Comprehensive quality evaluation model for underground space resources

The assessment and calculation of the comprehensive quality of underground space resources can reflect the comprehensive utilization value of underground space resources under the influence of geology, social economy and other conditions, which can be obtained by superimposing the difficulty and potential value of underground space development. The comprehensive quality can be expressed as:

4 $$ S = w_{d} S_{d} + w_{e} S_{e} $$

where w_d_ and w_e_ are the weights of the natural and socioeconomic factors, respectively; S_d_ and S_e_ are the resource development difficulty and potential value assessment value, respectively; and S is the overall quality.

### Evaluation index weight

**Figure 3:**
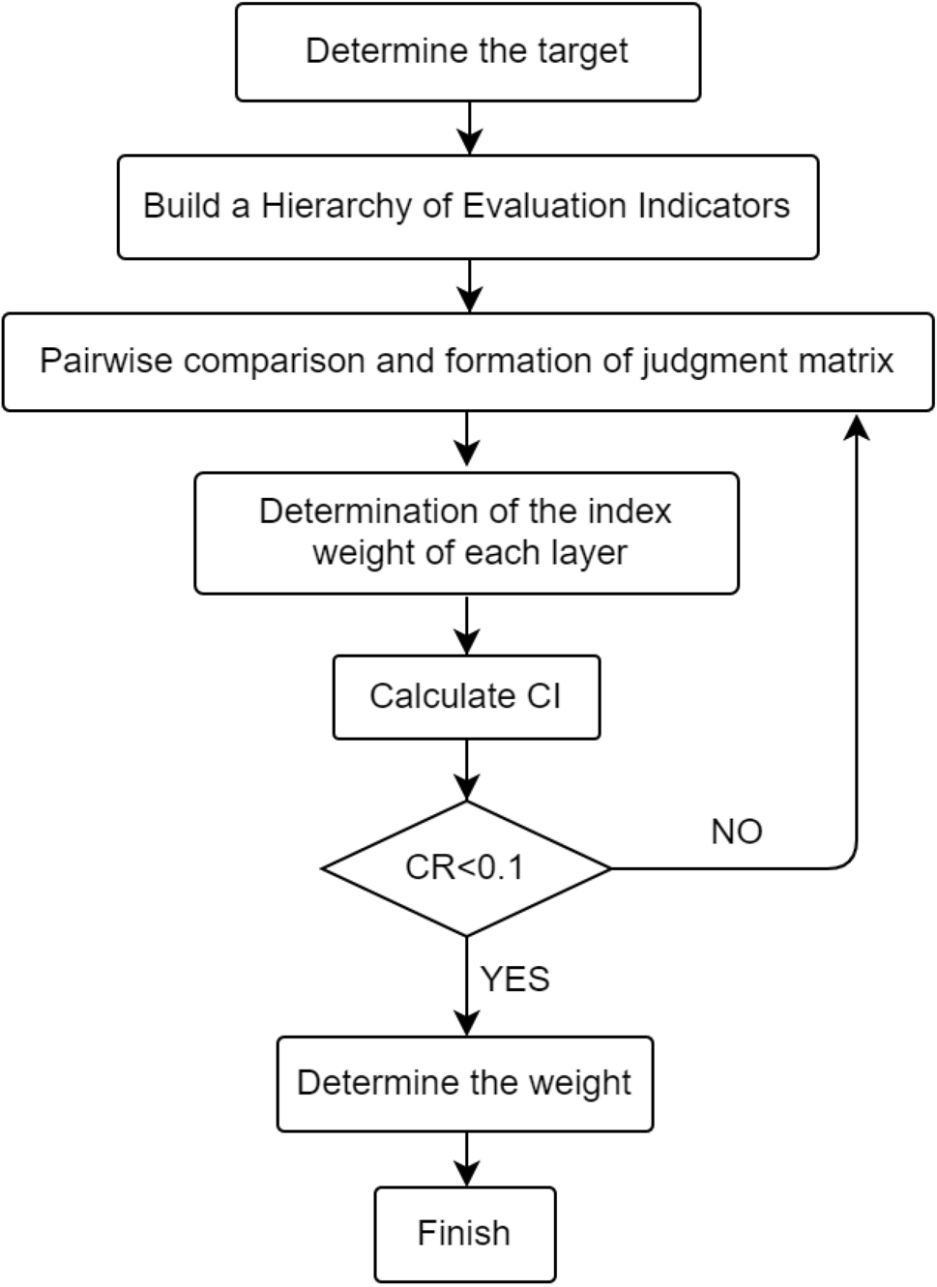
Flow chart of weight calculation using the AHP.

First, we compare the degrees of influence of the indicators to construct a judgement matrix and determine the relative importance of each pair of factors. This value is generally determined by the nine-point scaling method^34^. The weight of each indicator can be calculated by the arithmetic mean method in the analytical hierarchy process, as expressed in Eq. (5). The constructed comparison judgement matrix must be assessed for consistency. The criterion for determining whether the matrix meets the consistency requirement is CR < 0.1. For CR ≥ 0.1, the matrix does not meet the consistency requirements and must be rebuilt. CR can be calculated with Eq. (6). The weights of the indicators at all levels can be obtained according to the AHP flow chart, as shown in Fig. 3. The weights of all evaluation indicators are shown in Tables 3, 4, 5 and 6.

5 $$ \omega_{i} = \frac{1}{n}\sum\limits_{j = 1}^{n} {\frac{{a_{ij} }}{{\sum\nolimits_{k = 1}^{n} {a{}_{kj}} }}} $$

6 $$ CR = \frac{CI}{{RI}} $$

7 $$ \lambda_{\max } = \sum\limits_{i = 1}^{n} {\frac{{\sum\nolimits_{j = 1}^{n} {a_{ij} \omega_{j} } }}{{n\omega_{i} }}} $$

where $\omega_{i}$ is the index weight, $a_{ij}$ is the normalized value of the index in Row i and Column j, CI = ($\lambda_{\max }$ − n)/(n − 1), n denotes the order of the matrix, $\lambda_{\max }$ is the largest characteristic root of the matrix, which can be calculated according to Eq. (7), and RI is the average random consistency index.

**Table 6:** Comprehensive quality weight of underground space resources.

Indicator	Weight (0 ~ 15 m)	Weight (15 ~ 30 m)
Difficulty of developing underground space resources	0.2	0.3
Potential value of underground space resources	0.8	0.7

### Grading and quantification of the evaluation indicators

The evaluation indicators for the difficulty and potential value of underground space resource development are graded and quantified. The quantitative scores are selected from the [0, 1] interval according to the linear interpolation method. In this study, we divide each indicator into 4 levels, and the evaluation indicators are graded and scored. The first level is assigned a value of 1, the second level is assigned a value of 0.67, the third level is assigned a value of 0.33, and the fourth level is assigned a value of 0. According to the actual situation of Xianyang city, the scores of the different levels of evaluation indicators are shown in Tables 7 and 8.

**Table 7:** Difficulty index grading score of underground space resource development.

Theme layer	Indicator layer	Level 1 1	Level 2 0.67	Level 3 0.33	Level 4 0
Topography	Ground elevation/m	950 ~ 1200	1200 ~ 1500	1500 ~ 2000	> 2000
	Terrain slope/°	< 5	5 ~ 10	10 ~ 30	> 30
Bedrock and soil layer	Stratigraphic lithology	Sand interlayer	Sand	Clay/silt	Gravel
	Sand liquefaction	No liquefaction	Mild liquefaction	Moderate liquefaction	Severe liquefaction
Hydrogeology	Water content/(m^3^ d^−1^)	< 500	500 ~ 1000	1000 ~ 3000	> 3000
	Dive depth/m	< 15	10 ~ 15	5 ~ 10	< 5
Water resource protection	Secondary water source	Outside the water source			In the water source
Ecological sensitivity	Ecological spatial sensitivity	Nonsensitive area	City and county level	Provincial	national level
Existing buildings	Building type	Nonbuilt area	Low-rise building	Mid-rise building	High-rise building

**Table 8:** Grading and scoring of the potential value indicators for underground space resources.

Theme layer	Indicator layer	Level 1 1	Level 2 0.67	Level 3 0.33	Level 4 0
Thematic layer of socioeconomic conditions	Location conditions	Class I location	Class II location	Class III location	Class IV location
	Demographic situation/(10,000 people·km^−2^)	> 0.09	0.06 ~ 0.09	0.03 ~ 0.06	< 0.03
	Traffic conditions	Requirement level I	Requirement level II	Requirement level III	Requirement level IV
	Commercial benchmark land price/(yuan m^−2^)	> 4080	2700 ~ 4080	1800 ~ 2700	< 1800
	Urban land use type	Commercial finance and residential land	Traffic land	Industrial land	Ecological land and water bodies

### Grading of the evaluation results

With the use of the aforementioned evaluation model and method, based on the ArcGIS spatial analysis function, the single-factor layers are weighted and superimposed, and the evaluation results are graded by the principle of score interval evaluation and distribution (Table 9).

**Table 9:** Grading of the evaluation results.

Evaluation score	0.75 ~ 1	0.5 ~ 0.75	0.25 ~ 0.5	0 ~ 0.25
Development difficulty level	Low	Average	Higher	High
Potential value class	High	Higher	Average	Low
Comprehensive quality grade	High	Higher	Average	Low

## Evaluation results and analysis

### Estimation of the available resources for underground space development

With the use of ArcGIS statistics, excluding existing buildings, cultural relic reserves, ecological green space protection ranges, geological disaster-prone areas and water source protection areas in the main urban area of Xianyang city, the factors that control the underground space amount can be obtained, and the amount of development accounts for approximately 25.11% of the total development. According to Eq. (1) and Table 2, the underground space resources in shallow (0 ~ 15 m) and subshallow (15 ~ 30 m) areas in the main urban area of Xianyang city were estimated, and the underground space resources that could be reasonably developed under the different land types were obtained (Table 10).

**Table 10:** Amount of underground space resources available for rational development in the main urban area of Xianyang city (unit: 100 million m^3^).

Land use type	Shallow (0 ~ 15 m)	Subshallow (15 ~ 30 m)	Total	Proportion (%)
Roads	0.81	1.21	2.02	8.2
Green spaces and squares	1.2	1.5	2.7	11.0
Ground building	1.5	2.49	3.99	16.2
Water body	–	3.07	3.07	12.4
Farmland to be developed	5.72	7.15	12.87	52.2
total	9.23	15.42	24.65	100

The calculation results indicate that the current underground space development level in Xianyang city is still low, the exploitable area of the underground space in Xianyang city is approximately 82.3 km^2^, and the amount of shallow underground space in the main urban area of 30 m accounts for approximately 79.5% of the total underground space resources of the shallow and subshallow layers. However, underground buildings are different from surface buildings, with the characteristics of irreversible construction, and underground structures and the built environment often interact and influence each other. For example, underground structures at a certain development depth impose certain restrictions on the development of other structures at the same level. The difference in the degree of response of the different layers of different types of underground structures to seismic waves affects the interaction in development layer site selection. The current sufficient amount of development does not suggest that the underground space can be developed arbitrarily and subjectively, and unreasonable development can cause the proportion of the underground space that can be developed to be sharply reduced. Regarding underground transportation, the development of underground structures at unreasonable depths exerts a restrictive impact on the selection of underground transportation routes, reducing the amount of development along the traffic line.

### Assessment of the difficulty of underground space resource development

**Figure 4:**
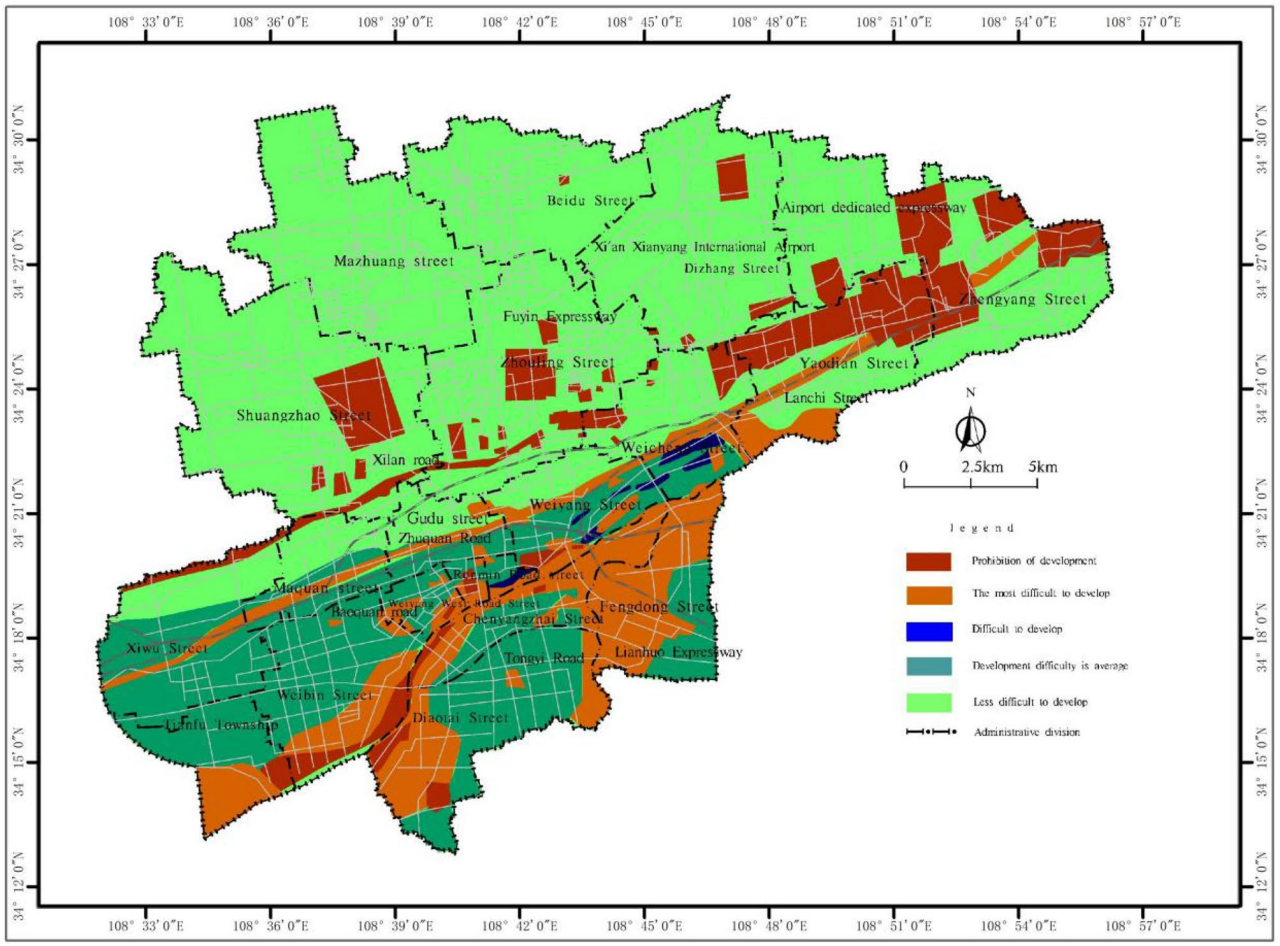
Distribution map of the difficulty distribution of shallow (0 ~ 15 m) underground space resource development in the main urban area of Xianyang city.

**Figure 5:**
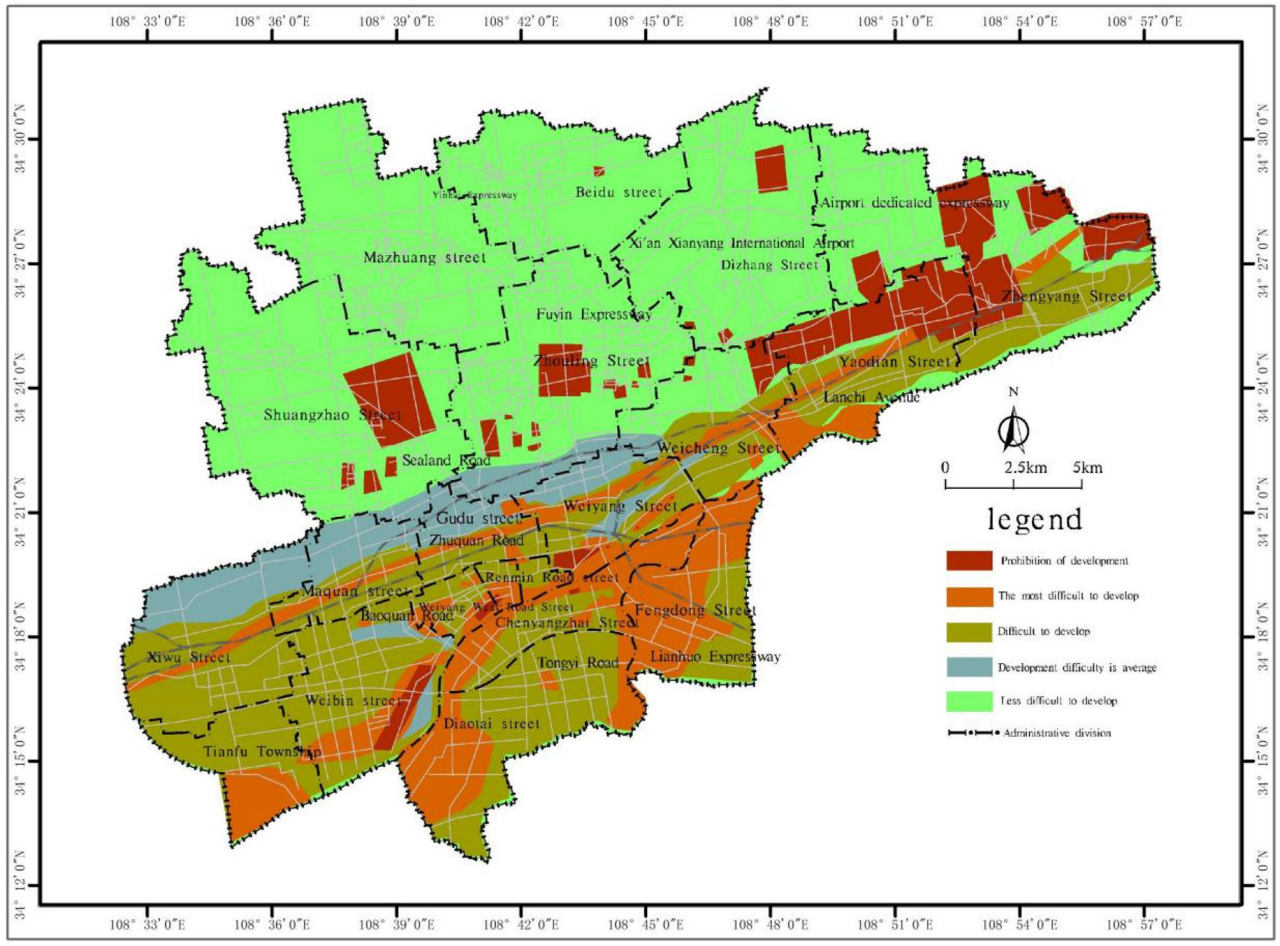
Distribution map of the difficulty of resource development of the subshallow (15 ~ 30 m) underground space in the main urban area of Xianyang city.

Based on the spatial analysis function in ArcGIS, the single-factor layers are superimposed to evaluate the development difficulty of shallow (0 ~ 15 m) and subshallow (15 ~ 30 m) underground space resources in the main urban area of Xianyang city, and a difficulty grading map of shallow to subshallow underground space resource development is generated (Figs. 4 and 5). Tables 11 and 12 provide the corresponding development difficulty grading descriptions.

**Table 11:** Grading description of the difficulty of resource development of the shallow (0 ~ 15 m) underground space in the main urban area of Xianyang city.

Development difficulty rating	Distribution area	Description	Area (km^2^)	% of the total
Prohibited area	Beiyuan cultural relic cemetery reserve	It is mainly limited by the distribution of cultural relics, ecological green spaces and water sources, it is located within the protection red line, and underground space development cannot occur in this area	67.76	11.6
The most difficult to develop	Along the active fault zone and its corresponding avoidance area	The main constraints of the engineering geological conditions are a low regional stability and highly water-rich aquifers. Some areas contain water sources and highways, and it is difficult to develop shallow resources	73.82	12.7
Difficult to develop	Some areas along the Weihe and Fenghe rivers	The engineering geological conditions are poor, and the main constraints include the serious problem of sand liquefaction, the high water richness of aquifers, and the difficulty of shallow resource development	2.60	0.5
The development difficulty is average	Part of the first and second terrace areas	The engineering geological conditions are general, the main constraint is the problem of loess collapse, sand liquefaction slightly impacts this area, and the high water richness of aquifers exerts a certain impact. The development of shallow resources is generally difficult	109.85	18.8
Less difficult to develop	Tertiary terrace	The engineering geological conditions are favourable, and the loess collapse problem is the main constraint of underground space development	329.07	56.4

**Table 12:** Grading description of the difficulty of resource development of the subshallow (15 ~ 30 m) underground space in the main urban area of Xianyang city.

Development difficulty rating	Distribution area	Description	Area (km^2^)	% of the total
Prohibited area	Beiyuan cultural relic cemetery reserve	It is mainly limited by the distribution of cultural relics and occurs within the red line of cultural relic protection, and ecological protection areas occur in this area, while railway underground space development is prohibited	54.21	9.3
The most difficult to develop	Along the active fault zone and its corresponding avoidance zone, water sources and high-speed arterial roads	The main constraints of the engineering geological conditions are a low regional stability and highly water-rich aquifers. Some areas are water sources and highways, and it is difficult to develop subshallow resources	72.34	12.4
Difficult to develop	Floodplain, first terrace, and second terrace area	The engineering geological conditions are poor, and the main constraints are extremely water-rich aquifers and slight sand liquefaction, and the development of subshallow resources is difficult	135.61	23.3
The development difficulty is average	Fenghe and Weihe first-class terraces, some areas of second-class terraces	The engineering geological conditions are general, and highly water-rich aquifers constitute the main problem in the development of the underground space. At the same time, the collapsibility of loess impacts the development of subshallow resources	49.67	8.5
Less difficult to develop	Tertiary terrace	The engineering geological conditions are favourable, and the problem of loess collapsibility is the main restrictive factor for the development of underground space	271.27	46.5

According to Figs. 4 and 5, the prohibition area in the main urban area of Xianyang city is mainly located at the southern edge of the Loess Plateau north of the Wei River, which is distributed along a northeast–east direction, mainly in the cultural relic cemetery protection area. The main urban area of Xianyang city is difficult to develop, and the general area occurs as a northeast–east band, concentrated in the low-terrace areas on both sides of the Wei River in the southern part of the main urban area, which are more difficult to develop in terms of underground space construction due to the large thickness of local sand and gravel layers and the complex hydrogeological conditions. The areas with low development difficulties in the main urban area of Xianyang city are mainly distributed in the loess plateau area in the northern part of the city, the thick loess layer exhibits suitable engineering geological characteristics, and the difficulty of underground space development and construction is relatively low.

As an urban environmental geotechnical engineering activity, the development of urban underground space resources is difficult due to regional engineering geological conditions and urban environmental constraints. According to Tables 11 and 12, the difficulty of resource development of the shallow underground space in the main urban area of Xianyang city is affected by rock and soil problems, as well as the constraints of ecological protection requirements and existing building facilities, and the difficulty of resource development of the subshallow underground space is relatively high, mainly reflected in the obvious impact of groundwater on underground space development. In general, with increasing development level, the development difficulty correspondingly increases, and it is mainly controlled by the geological conditions.

### Evaluation of the potential value of underground space resources

**Figure 6:**
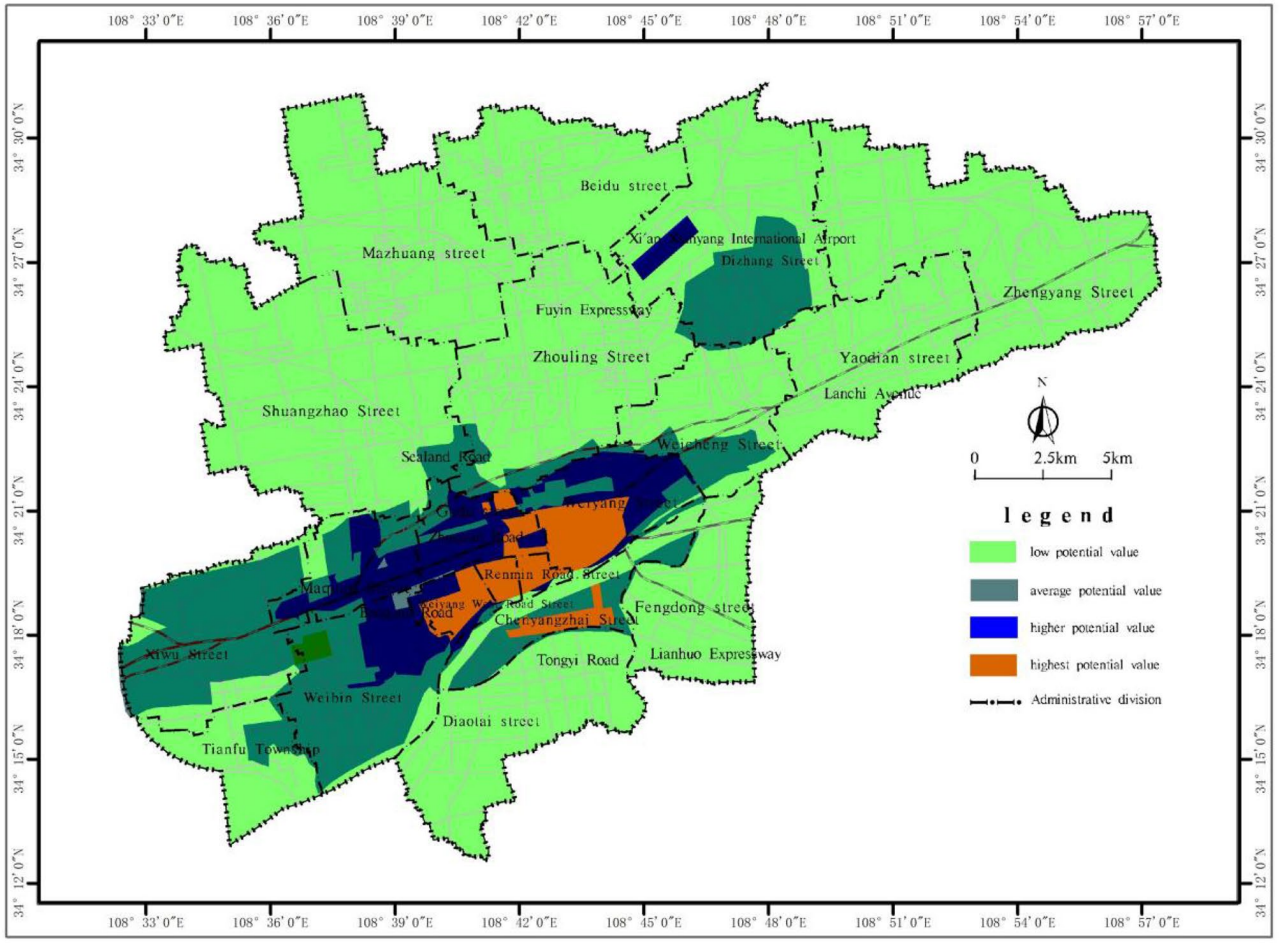
Classification map of the potential value of the underground space resources in the main urban area of Xianyang city.

By weighting and integrating the distribution of social factors such as location, population, economy, transportation, and land use type in the main urban area of Xianyang city, a potential value distribution map of the underground space in the main urban area of Xianyang city is obtained (Fig. 6).

As shown in Fig. 6, the area with a high potential value of the underground space in the main urban area of Xianyang city is the expansion area around the core area and the core area on the south bank of the Wei River, which exhibits the highest degree of development in terms of the population density, economic level, transportation and location conditions. The area involved covers 17.99 km^2^, of which the area north of the Wei River is the expansion of the core area, mainly involving residential and commercial areas around the urban core area, while the area south of the Wei River is mainly distributed at the centre of Century Avenue. The area was developed late, the planning concept is relatively new, and it provides a higher development potential by virtue of the advantages of its geographical location. The area with a high potential value mainly involves residential and industrial land around the northern urban area of Weihe, covering a total of 32.31 km^2^, affected by the distribution of industrial plants and railway barriers. The area has a low population density and lacks large-scale commercial office facilities. Considering the parking and civil defence needs of the residential area and storage use in the industrial zone, the area still exhibits high development potential. The area with a general potential value is mainly located in the built-up area north of the Wei River, the southern extension area of the Wei River and the surrounding area of the airport, covering a total of 95.05 km^2^. The area has not yet been built up on a large scale, and many areas are vacant. Considering that the future development plan Xianyang mainly aims to expand the city to these areas, there is also a certain development value of the underground space. The area with a low potential value involves the 437.75 km^2^ area outside the built-up area of the city, which has not yet been developed on a large scale except for the airport area. The land mostly encompasses farmland and cultural relic sites, and the development value is low.

### Comprehensive quality evaluation of underground space resources

**Figure 7:**
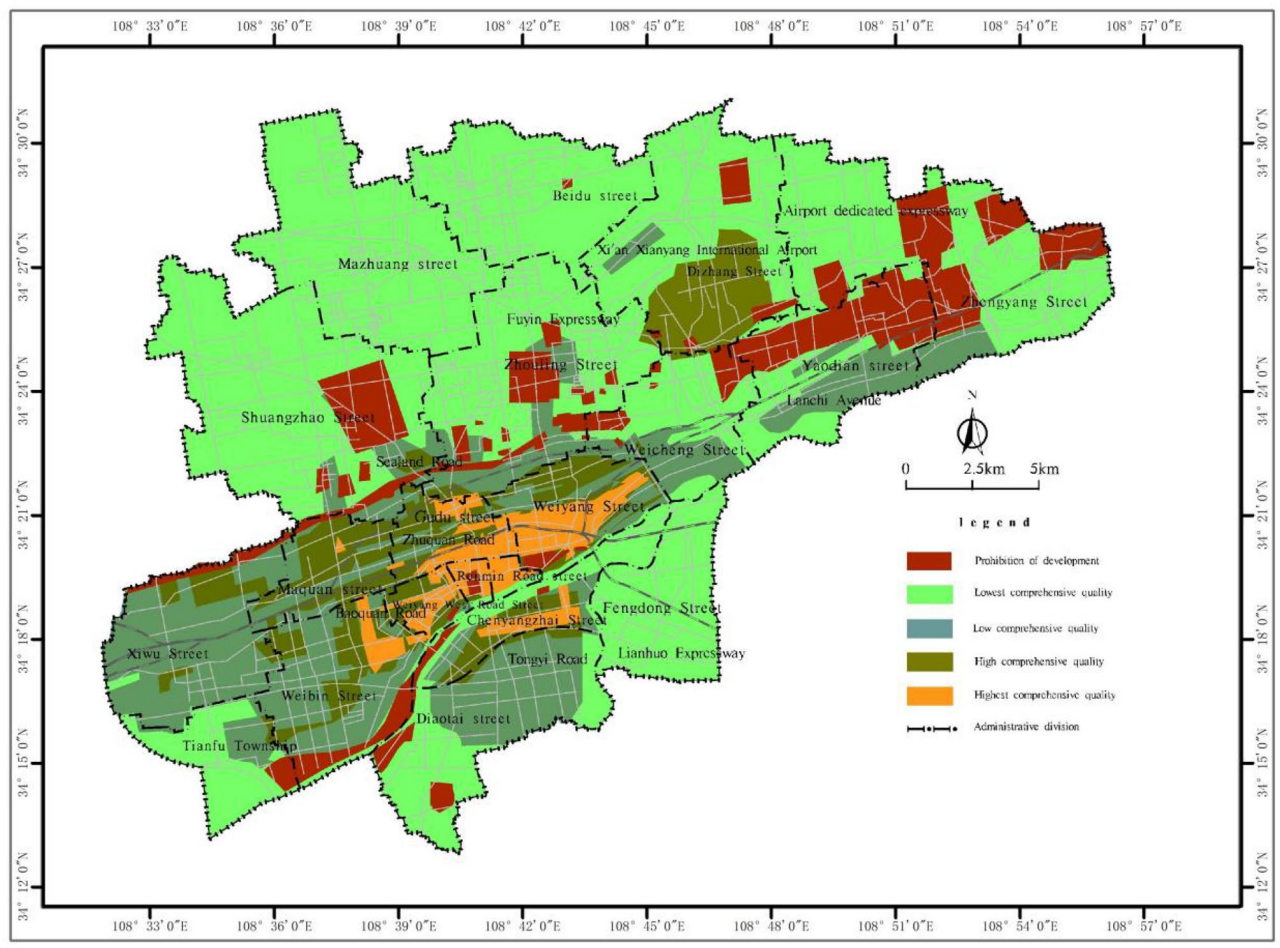
Comprehensive quality grading map of the shallow (0 ~ 15 m) underground space resources in the main urban area of Xianyang city.

**Figure 8:**
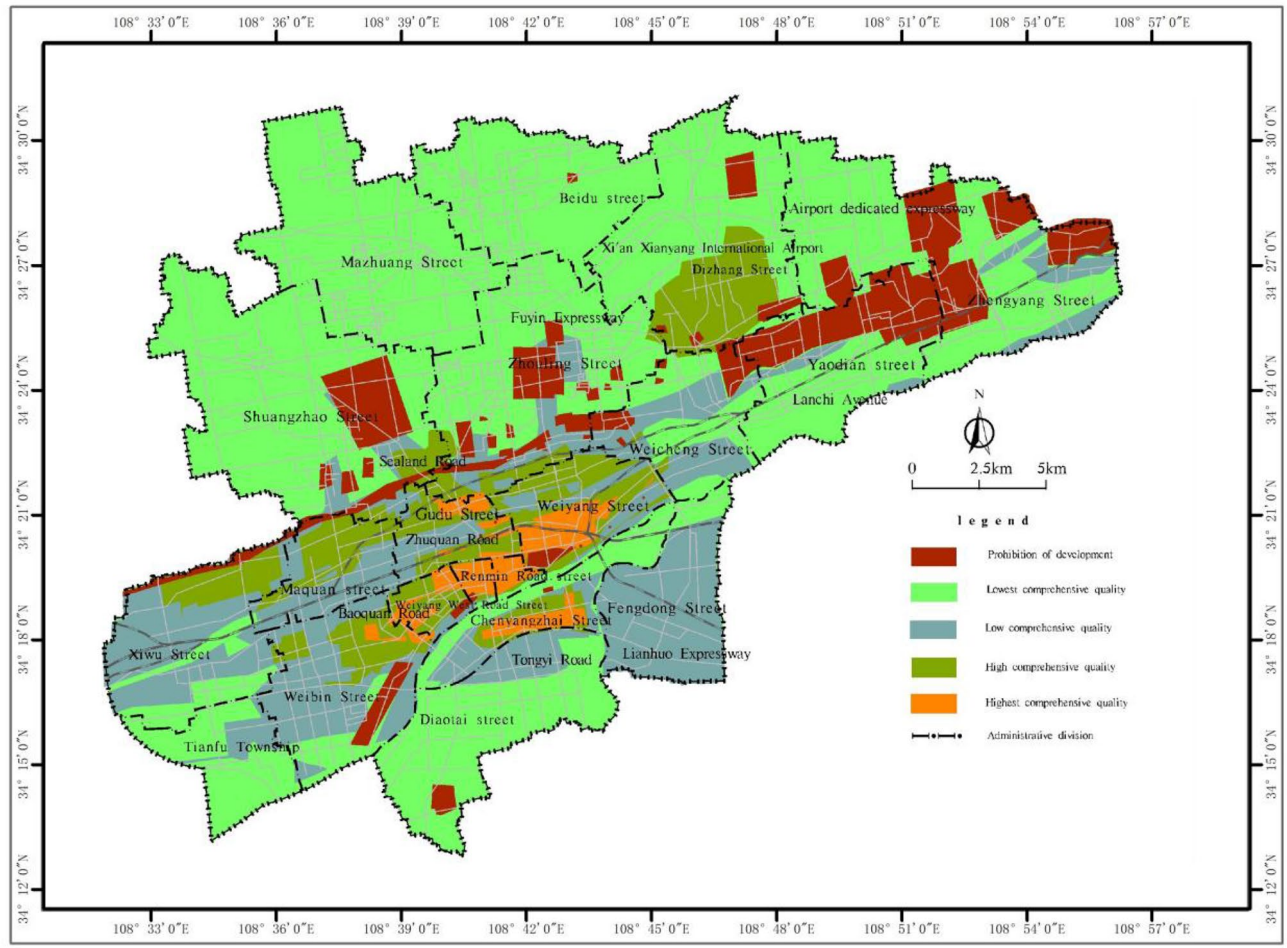
Comprehensive quality grading map of the subshallow (15 ~ 30 m) underground space resources in the main urban area of Xianyang city.

With the use of the evaluation model and method described above, based on the spatial analysis function in ArcGIS, the development difficulty and potential value of underground space resources in the main urban area of Xianyang city were superimposed to generate a comprehensive quality grading map of the shallow to subshallow underground space resources (Figs. 7 and 8).

The comprehensive quality grading map of the shallow to subshallow underground space resources in Xianyang city clearly reflects the comprehensive resource endowment in the main urban area, among which the core areas such as Renmin Road, Weiyang Road and Century Avenue cover a total area of 21.52 km^2^, and the comprehensive quality grade of the underground resources in the shallow area (0 ~ 15 m) is the highest, while the area with the highest grade in the subshallow area (15 ~ 30 m) is reduced to 4.37 km^2^ due to the increase in construction difficulty. The comprehensive quality grade of the underground resources in the surrounding extension area of the urban core area and the airport area is high, and the shallow and subshallow areas are 55.98 km^2^ and 65.49 km^2^, respectively. Affected by the adverse geological conditions and location distribution, the comprehensive quality grade of the core area and the extended area around the Weihe River is low, and the areas in the shallow and subshallow areas under this grade are 110.62 km^2^ and 112.32 km^2^, respectively. The comprehensive quality grade of the underground space resources in the Beiyuan area except the airport and other surrounding areas is the lowest, and the areas in the shallow and subshallow areas are 327.22 km^2^ and 346.71 km^2^, respectively. The cultural relic sites, water source protection areas and ecological reserves in the planning area are prohibited development areas due to the requirements of relevant norms and protection, with a shallow restricted area of 67.76 km^2^ and a subshallow restricted area of 54.21 km^2^.

## Discussion

The development of the underground space in the main urban area of Xianyang city is in its infancy, and the amount of underground space resources available for development and utilization is considerable. According to the difficulty and potential value at each level of development, the comprehensive quality at the different levels is considered in an overall manner, and the rational selection and planning of development locations can greatly improve the efficiency of underground space utilization. From the perspective of the development difficulty, it is necessary to consider the comprehensive geological conditions of the region, which can affect the rationality, safety and economy of underground space development, construction, operation and management. From the perspective of the potential value, the population density, economic conditions, urban functional layout and location conditions undoubtedly have different demands for underground space development and construction, which affects the social value and operational benefits of underground space development. On the basis of considering the comprehensive quality at the different levels, development sequence determination and location selection of regional underground space locations can be achieved.

In the main urban area of Xianyang city, the comprehensive quality level of shallow underground space development is general and above, reaching approximately 188.12 km^2^, accounting for 32.26% of the total quantity, and the comprehensive quality of the underground space resources of Weiyang West Road, Renmin Road Street and Weiyang Street in Xianyang city is very high, which exhibits both the urgency of development and favourable geological conditions. There are slightly better geological conditions and some areas with a higher demand in the northeastern area of Maquan Street, part of Gudu Street and Xixian International Airport in Dizhang Street, so the comprehensive quality of resources in this area is also high, and the above areas can be considered during shallow development.

The comprehensive quality level of subshallow underground space development in the main urban area of Xianyang city is general and above, reaching approximately 182.18 km^2^, accounting for 31.24% of the total quantity, and the comprehensive quality of the underground space resources of Weiyang West Road, Renmin Road Street and Weiyang Street in Xianyang city remains high, but due to geological environment problems, subshallow development is impacted. There are favourable geological conditions and areas with certain needs in the northeast of Xiwu Street, the northern area of Maquan Street, some areas of Gudu Street and Xixian International Airport in Dizhang Street, so the comprehensive quality of the resources in this area is also high, and subshallow development should be considered and planned in these areas.

## Conclusion

There are many factors that affect the comprehensive evaluation of urban underground space resources, and these factors are multilevel and complex. In this paper, we used the analytic hierarchy process to comprehensively evaluate underground space resources as a whole, organically combining qualitative and quantitative methods. We decomposed complex systems and transformed multicriteria decision-making problems that are difficult to fully quantify into multilevel single-objective problems for obtaining reasonable and clear evaluation process levels, and the results were simple and clear. Regarding comprehensive evaluation of the underground space resources in the main urban area of Xianyang city, the following conclusions can be drawn.

In terms of quantity, at present, the underground space resources available for development in the main urban area of Xianyang city account for approximately 25.11% of the total development quantity, the underground developable area is approximately 82.3 km^2^, and the shallow underground space resources (30 m) available for development in the main urban area reach 2.465 billion m^3^, accounting for approximately 79.5% of the total resources of the shallow underground space. The potential for development and utilization is enormous, underground space development mainly involves shallow depths, the degree of subshallow development is low, and the underground space development level is still relatively low.

In terms of the comprehensive quality, first, the main urban area of Xianyang city was evaluated in regard to the difficulty of underground space resource development, and the notable influences of the topography and geomorphology, rock and soil mass, structural stability and groundwater occurrence characteristics on the development of the underground space at different depths in Xianyang were studied and summarized. The specific role and spatial characteristics of the impact distribution were determined. The development of subshallow underground space resources is relatively difficult, mainly reflected in the obvious impact of groundwater on underground space development. Second, based on socioeconomic influencing factors such as population, traffic conditions, location conditions, land price and land use type, the potential value of underground space resources was evaluated, and the potential value distribution of the underground space in the study area was obtained. The areas with a high potential value were the areas along Renmin Middle Road and the middle of Weiyang Road, the core area of the south bank of the Wei River, and the residential and industrial land areas around the northern urban area of the Wei River, while the other regional areas exhibited a low degree of development and low potential value. Finally, according to the evaluation results for the difficulty of underground space resource development and the evaluation results for the potential value of underground space resources, the comprehensive quality of shallow and subshallow underground space resources in the main urban area of Xianyang city was graded. The comprehensive quality level of the underground space resources in core areas such as Renmin Road, Weiyang Road and Century Avenue was the highest, the comprehensive quality of the underground space resources in the expansion area around the urban core area and the airport area was the second highest, and the comprehensive quality level in the area outside the core area and the extension area around the Weihe River was average. The comprehensive quality level of the underground space resources in the Beiyuan area, except for the airport area and other surrounding areas, was low.

## Data Availability

Data are available upon reasonable request, please contact the corresponding author.
